# Typing of Panton-Valentine Leukocidin-Encoding Phages and *lukSF-PV* Gene Sequence Variation in *Staphylococcus aureus* from China

**DOI:** 10.3389/fmicb.2016.01200

**Published:** 2016-08-03

**Authors:** Huanqiang Zhao, Fupin Hu, Shu Jin, Xiaogang Xu, Yuhan Zou, Baixing Ding, Chunyan He, Fang Gong, Qingzhong Liu

**Affiliations:** ^1^Department of Clinical Laboratory, Shanghai General Hospital, School of Medicine, Shanghai Jiaotong UniversityShanghai, China; ^2^Institute of Antibiotics, Huashan Hospital, Fudan UniversityShanghai, China; ^3^Experimental Research Center, Shanghai People's Hospital of Putuo DistrictShanghai, China; ^4^Department of Clinical Laboratory, The Third Hospital Affiliated to Nantong UniversityWuxi, China

**Keywords:** *Staphylococcus aureus*, Panton-Valentine leukocidin, sequence variation, phage typing, genetic background

## Abstract

Panton-Valentine leukocidin (PVL, encoded by *lukSF-PV* genes), a bi-component and pore-forming toxin, is carried by different staphylococcal bacteriophages. The prevalence of PVL in *Staphylococcus aureus* has been reported around the globe. However, the data on PVL-encoding phage types, *lukSF-PV* gene variation and chromosomal phage insertion sites for PVL-positive *S. aureus* are limited, especially in China. In order to obtain a more complete understanding of the molecular epidemiology of PVL-positive *S. aureus*, an integrated and modified PCR-based scheme was applied to detect the PVL-encoding phage types. Phage insertion locus and the *lukSF-PV* variant were determined by PCR and sequencing. Meanwhile, the genetic background was characterized by staphylococcal cassette chromosome *mec* (SCC*mec*) typing, staphylococcal protein A (*spa*) gene polymorphisms typing, pulsed-field gel electrophoresis (PFGE) typing, accessory gene regulator (*agr*) locus typing and multilocus sequence typing (MLST). Seventy eight (78/1175, 6.6%) isolates possessed the *lukSF-PV* genes and 59.0% (46/78) of PVL-positive strains belonged to CC59 lineage. Eight known different PVL-encoding phage types were detected, and Φ7247PVL/ΦST5967PVL (*n* = 13) and ΦPVL (*n* = 12) were the most prevalent among them. While 25 (25/78, 32.1%) isolates, belonging to ST30, and ST59 clones, were unable to be typed by the modified PCR-based scheme. Single nucleotide polymorphisms (SNPs) were identified at five locations in the *lukSF-PV* genes, two of which were non-synonymous. Maximum-likelihood tree analysis of attachment sites sequences detected six SNP profiles for *attR* and eight for *attL*, respectively. In conclusion, the PVL-positive *S. aureus* mainly harbored Φ7247PVL/ΦST5967PVL and ΦPVL in the regions studied. *lukSF-PV* gene sequences, PVL-encoding phages, and phage insertion locus generally varied with lineages. Moreover, PVL-positive clones that have emerged worldwide likely carry distinct phages.

## Introduction

*Staphylococcus aureus* causes a spectrum of diseases from minor skin and soft tissue infections (SSTIs) to life-threatening conditions due to its potential to produce many toxins and efficiency at overcoming antibiotics (David and Daum, 2010; Uhlemann et al., 2014). Panton-Valentine leukocidin (PVL), found in some strains of *S. aureus*, plays a key role in the leukocytolysis and tissue necrosis (Shallcross et al., 2013). The genes (*lukSF-PV*) encoding PVL are composed of two co-transcribed open reading frames (*lukS-PV* and *lukF-PV*), and located on lysogenized bacteriophages integrated into *S. aureus* chromosome (Boyle-Vavra and Daum, 2007; Shallcross et al., 2013).

To date, all known *S. aureus* phages belong to the order *Caudovirales*, which can be separated into three major families (*Podoviridae, Siphoviridae*, and *Myoviridae*) depending on the tail morphology (Xia and Wolz, 2014). At least 10 PVL phages have been described and sequenced, and all of them belong to the *Siphoviridae* family characterized by double-stranded DNA and a long non-contractile tail (Zhang et al., 2011; Xia and Wolz, 2014). As the mobile genetic elements, phages can be mobilized and transferred among *S. aureus* strains. Hence, investigating the typing of PVL-encoding phages among *S. aureus* may help to better understand the evolution of these pathogens. The PCR-based scheme targeting a small part of the phage genome is a cursory means of predicting phages types inexpensively. However, there is no unified pattern for this scheme. To identify as many PVL-encoding phages as possible, this article summarized a strategy by integrating and modifying the expanded PCR-based scheme described previously (Ma et al., 2008; Boakes et al., 2011; Chen et al., 2013; Sanchini et al., 2014). Subsequently, by the usage of this strategy, fifteen-reaction PCR assay was carried out to detect 10 of the PVL-encoding phages (ΦPVL, Φ108PVL, Φtp310-1, ΦSa2958, ΦSa2mw, ΦSLT, ΦSa2USA, ΦTCH60, Φ7247PVL/ ΦST5967PVL, and ΦSa119) in *S. aureus* from China.

At least 22 single-nucleotide polymorphisms (SNPs) have been identified in the *lukSF-PV* genes (Wolter et al., 2007; O'Hara et al., 2008; Boakes et al., 2011; Li et al., 2012; Chen et al., 2013; Sanchini et al., 2014). And several PVL protein isoforms carrying non-synonymous mutations have recently been revealed (O'Hara et al., 2008; Li et al., 2012), which may implicate functional significance. As we all know, phages lysogenize into the bacterial chromosome through the integrative pathway. Boakes et al. (2011) analyzed the sequence diversity at the insertion site for the different PVL-encoding phages and found two lineage-specific insertion sites within the *S. aureus* chromosome: Ins1 and Ins2. Of note, site-specific attachment sequences (*attL* and *attR*) are also conserved across lineages of PVL-carrying strains although some variations were found (Boakes et al., 2011; Chen et al., 2013).

Due to the absence of a uniform detection method, complete data on Chinese epidemiology of PVL-encoding phages are limited (Li et al., 2012; Hu et al., 2015). In addition, no details yet about chromosomal PVL-encoding phage insertion sites for *S. aureus* in China are reported. In the present study, we aimed to obtain a more complete description of the molecular epidemiology of PVL-positive *S. aureus* from China by detecting PVL-encoding phage types, analyzing PVL variant alleles and the chromosomal phage insertion junctions, and determining the genetic background. Moreover, the relationships among them were also elucidated.

## Materials and methods

### Bacterial isolates

**Figure 1:**
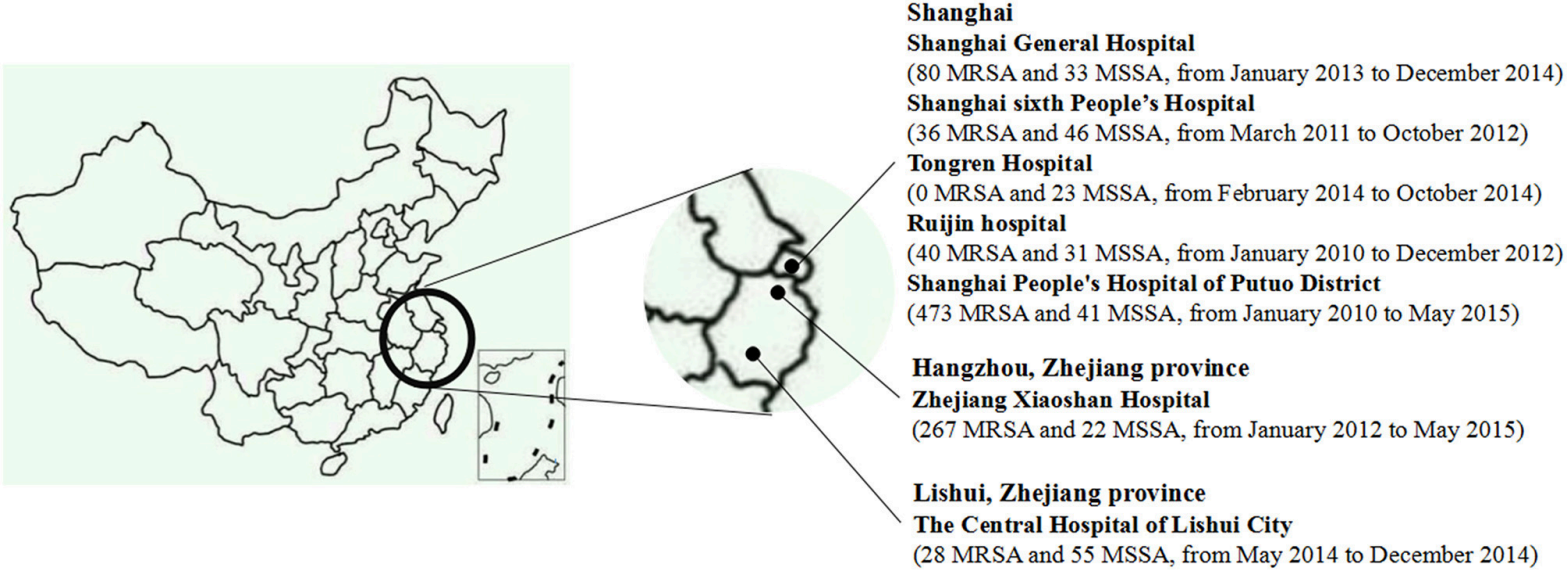
**The geographic distribution of 1175 ***S. aureus*** isolates**.

During January 2010 to May 2015, a total of 1175 consecutive, non-duplicate clinical *S. aureus* isolates were collected from seven hospitals in China, namely, Shanghai General Hospital, Shanghai sixth People's Hospital, Tongren Hospital, Ruijin hospital, Shanghai People's Hospital of Putuo District, Zhejiang Xiaoshan Hospital, and The Central Hospital of Lishui City, Zhejiang province (Figure 1). All the isolates, including 924 methicillin-resistant *S. aureus* (MRSA) strains and 251 methicillin-susceptible *S. aureus* (MSSA) strains, were identified by VITEK Systems (BioMérieux, Marcy l′ Etoile, France), the susceptibility of cefoxitin (30 μg, Oxoid, Basingstoke, UK), and the presence of *mecA* and *mecC* genes (Bignardi et al., 1996; Ganesan et al., 2013; Clinical Laboratory Standards Institute, 2014). The presence of *lukSF-PV* genes was determined by PCR according to previously published method (Lina et al., 1999).

### Single-nucleotide polymorphisms of the *lukSF-PV* genes

All isolates were cultured on blood agar and incubated overnight at 37°C. Genomic DNA was extracted by TIANamp Bacterial DNA Kit (TIANGEN BIOTECH Co., Ltd., Beijing, China). Three primer pairs were designed to amplify three fragments (654, 718, and 680 nucleotides in length, respectively) of *lukSF-PV* genes as described by Boakes et al. (2011). All the products of PCR were sequenced in both directions by Shanghai Sangon Biotech.

### Characterization of PVL-positive isolates

PVL-positive *S. aureus* were characterized by staphylococcal cassette chromosome *mec* (SCC*mec*) typing (Zhang et al., 2005), staphylococcal protein A (*spa*) gene polymorphisms typing (Koreen et al., 2004), pulsed-field gel electrophoresis (PFGE) typing (Mulvey et al., 2001), accessory gene regulator (*agr*) locus typing (Lina et al., 2003) and multilocus sequence typing (MLST; Enright et al., 2000). The *spa* type for each isolate was obtained based on a website (http://www.ridom.de/spaserver/). Sequence types (STs) were determined by allelic profile according to the MLST database (http://saureus.mlst.net/). And clustering of related STs into clonal complexes (CCs) was analyzed using eBURST (http://www.mlst.net).

### PVL-encoding phage typing

**Figure 2:**
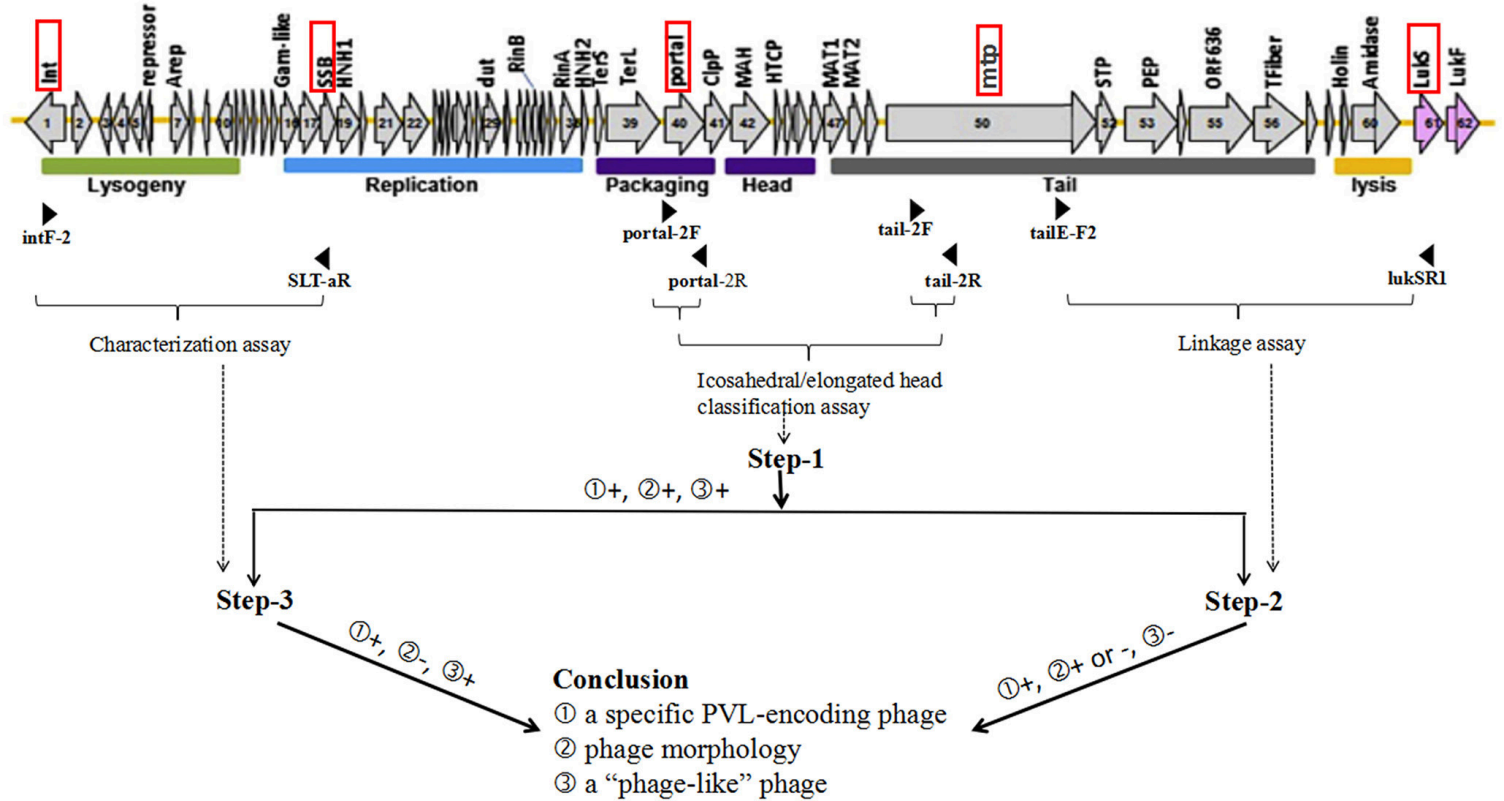
**Work flow for PVL-encoding phage typing**. The structure of ΦSLT (GenBank accession no. AB045978) (Xia and Wolz, 2014) and three steps of set-2 PCRs are taken as examples for illustrating the workflow for determination of PVL-encoding phages. The red rectangular boxes indicate target loci of phage typing primers, and the positions of primers are indicated by black arrowheads. Horizontal braces indicate the PCR or PCR groups for 3 steps of PCR-based scheme. The dotted arrows represent the correspondence between assays and steps. Figures in the circle represent several possibilities for PCR results or conclusions. “+” indicates positive result, while “–” indicates negative result.

Integrating the expanded PCR-based scheme described by previous studies (Ma et al., 2008; Boakes et al., 2011; Chen et al., 2013; Sanchini et al., 2014) with a little modification, three sets of PCRs including 15 PCR reactions (Set 1: PCR-1, -4 -7, and -8, Set 2: PCR-2, -5, and -9 to -13, and Set 3: PCR-3, -6, -14, and -15), were carried out to detect 10 of the PVL-encoding phages (ΦPVL, Φ108PVL, Φtp310-1, ΦSa2958, ΦSa2mw, ΦSLT, ΦSa2USA, ΦTCH60, Φ7247PVL/ ΦST5967PVL, and ΦSa119) (Table 1). PCR-1 to -3 (step-1) targeting genes encoding icosahedral or elongated head shape allowed classification of elongated-head group, icosahedral-head group I and icosahedral-head group II phages; PCR-4 to -6 (step-2) were used to link these morphologically specific tail genes to the *lukSF-PV* genes with primer pairs commonly conserved among each of the groups; PCR-7 to -15 (step-3) were designed to detect 10 specific PVL-encoding phages. All the PVL-positive isolates were detected by PCR-1 to -3 (step-1). Only when a positive result was obtained from step-1, were step-2, and step-3 performed. Phages which were positive by icosahedral/elongated head classification assays (PCR-1 to -3) and characterization assays (PCR-7 to -15) but negative for linkage assays (PCR-4 and -6), were defined as “phage-like” depending on the existence of known individual phage type (Chen et al., 2013). The workflow and the detailed PCR-based scheme for PVL-encoding phage typing were summarized in Figure 2 and Table 1, respectively.

**Table 1:** **PCR-based scheme for PVL-phage typing**.

**Step 1**							**Step 2**							**Step 3**						
	**Primer use**	**Primer name**	**Target locus**	**Size of PCR product (bp)**	**References**	**PCR's order number**	**Primer use**	**Primer name**	**Target locus**	**Size of PCR product (bp)**	**References**	**PCR's order number**	**PVL phage(s)**	**Primer use**	**Primer (pair) name**	**Target locus**	**Size of PCR product (bp)**	**GenBank accession no**.	**References**	**PCR's order number**
Set 1	Icosahedral-head group I	portal-1F	*por*	569	Ma et al., 2008	PCR-1	To link icosahedral-head group I specific tail genes to the PVL genes.	tail-ico-F	*mtp*	8570	Ma et al., 2008	PCR-4	ΦPVL	To Classify individual PVL phages by detecting the gene lineage between the integrase gene and the genes located downstream of the gene.	intF-2/PVL-aR	*int*/JP030	1411	AB009866	Ma et al., 2008	PCR-7
		portal-1R	*por*		Ma et al., 2008					10,497			Φ108PVL		intF-2/108-aR	*int*/*ant*	4340	AB243556	Ma et al., 2008	PCR-8
		tail-1F	*mtp*	489	Ma et al., 2008			lukSR1	*lukS-PV*	8574	Ma et al., 2008		Φtp310-1^*^		intF-2/PVL-aR /108-aR^*^	*int*/JP030/ *ant*	1411/4579^*^	EF462197	Ma et al., 2008; Chen et al., 2013	PCR-7, -8
		tail-1R	*mtp*		Ma et al., 2008														
Set 2	Elongated-head group	portal-2F	*por*	656	Ma et al., 2008	PCR-2	To link elongated-head group specific tail genes to the PVL genes.	tailE-F2	*mtp*	9483	Otter et al., 2010	PCR-5	ΦSa2958	To Classify individual PVL phages by detecting the gene lineage between the integrase gene and the genes located downstream of the gene.	intF-2/2958-aR	*int*/JP004	2238	AP009363	Ma et al., 2008	PCR-9
		portal-2R	*por*		Ma et al., 2008					9484			ΦSa2mw		intF-2/MW2-aR	*int*/ *cro*	4065	BA000033	Ma et al., 2008	PCR-10
		tail-2F	*mtp*	468	Ma et al., 2008			lukSR1	*lukS-PV*	9486	Ma et al., 2008		ΦSLT		intF-2/SLT-aR	*int*/ *ssb*	8770	AB045978	Ma et al., 2008	PCR-11
		tail-2R	*mtp*		Ma et al., 2008				9484			ΦSa2USA	Sa2USA-F /Sa2USA-R2		*phiSLT ORF484-like/lukS-PV*	679	CP000255	Boakes et al., 2011; Sanchini et al., 2014	PCR-12
										9482			ΦTCH60		intF-2/ TCH60-aR	*int*/*HMPREF0772-11656*	2675	NC-017342	Ma et al., 2008; Chen et al., 2013	PCR-13
Set 3	Icosahedral-head group II	portal-3F	*por*	535	Sanchini et al., 2014	PCR-3	To link icosahedral-head group II specific tail genes to the PVL genes.	TAIL-5	*mtp*	9164	Sanchini et al., 2014	PCR-6	Φ7247PVL/ΦST5967PVL	To Classify individual PVL phages by detecting the gene lineage between the integrase gene and the genes located downstream of the gene.	intF-2/repR	*int/rep*	2965	AP011956 /AP011955	Ma et al., 2008; Chen et al., 2013; Sanchini et al., 2014	PCR-14
		portal-3R	*por*																	
		tail-3F	*mtp*	842	Sanchini et al., 2014			lukSR1	*lukS-PV*	10.728	Ma et al., 2008		ΦSa119		intF-2/SA119Ant1	*int/ant*	4918	KJ596420	Ma et al., 2008; Sanchini et al., 2014	PCR-15
		tail-3R	*mtp*																	

* *Φtp310-1 harbors an icosahedral head and can be identified by the presence of PCR products specific both for ΦPVL and Φ108PVL (positive both for PCR-7 and -8)*.

In case of the fragments amplified >3000 nucleotides, the PCRs were carried out using PrimeSTAR® Max DNA Polymerase (TaKaRa, Dalian, China) adhering to the protocols recommended by manufacturer.

### Phage insertion locus sequencing

PCR designed to detect the proximal and distal junctions of PVL-encoding phage insertion sites were performed by the method described previously (Boakes et al., 2011). Sequences were aligned using CLUSTA L X 2.0. Phylip3.6 was used to construct a maximum-likelihood phylogenetic tree with 500 bootstrap replicates for the junction region sequences.

## Results

### Frequency and characterization of *S. aureus* harboring *lukSF-PV* genes

Of 1175 *S. aureus* isolates, 78 (6.6%) isolates were *lukSF-PV* genes positive, including 62 MRSA and 16 MSSA. Among the 78 *lukSF-PV* genes positive strains, 66 (55 MRSA and 11 MSSA) were *agr*1, 9 (6 MRSA and 3 MSSA) were *agr*3, and 2 (MSSA) were *agr*4. One isolate was unable to be classified in any of the established *agr* groups, which may be an *agr*-negative variant associated with the extensive use of antibiotics (Paulander et al., 2013). Of course, point mutation occurring in primer binding sequence may be another reason. MLST results showed 15 ST types were produced in isolates studied, namely ST1 (CC1), ST9 (CC9), ST22 (CC22), ST25 (CC25), ST30 (CC30), ST59 (CC59), ST88 (CC88), ST188 (CC1), ST149 (CC5), ST217 (CC22), ST338 (CC59), ST398 (CC398), ST1301 (CC121), ST160 (CC121), and ST172 (singleton). ST59 (64.5%, 40/62) was the most frequent ST in MRSA isolates, while ST398 (25%, 4/16) was the most prevalent type in MSSA strains. Two STs identified in this study have not found matching profiles in the MLST database, and subsequently were designated ST160 and ST172 after we uploaded the data to the website (http://pubmlst.org/saureus/). By sequence analysis of PCR products of the *spa* gene, 23 *spa* types were yielded in 78 PVL-positive isolates. The most common *spa* type identified was t437 (48.7%, 38/78). Two *spa* types determined in this study were not found in Ridom SpaServer, which were subsequently designated t15796 and t15797 after our submission. It is notable that 94.7% (36/38) of t437 isolates were associated with CC59. In addition, most of t034 strains (75%, 3/4) related to CC398. PFGE typing showed that 78 PVL-positive isolates were divided into 17 patterns. Most of isolates were clustered into PFGE type A (subtypes A1 to A7), type B (subtypes B1 to B3), and type C (subtypes C1 to C7) together accounting for 44.9% (35/78). In 62 PVL-positive MRSA isolates, 57 could be typed (SCC*mec* I-V) by SCC*mec* typing method, and 5 (8.1%) were non-typeable. The SCC*mec* type most commonly found was type III (53.2%, 33/62), followed by type IVa (32.3%, 20/62), type II (3.2%, 2/62), type IVb, and type V (1.6%, 1/62 each). The detailed molecular characterization of PVL-positive isolates by SCC*mec, agr, spa*, PFGE, and MLST typing was shown in Table 2.

**Table 2:** **Characteristics and PVL-phage typing of 78 PVL-positive ***S. aureus*** isolates**.

**Isolate ID**	**location**	**ST**	**CC**	**SCC*mec***	***spa* type**	***agr* type**	**PFGE**	***attL* cluster**	***attR* cluster**	**Phage morphology**	**PVL-phage type**	**Isoform of PVL**
**MRSA (*****n*** = **62)**												
7	Zhejinag	ST1	CC1	V	t114	I	K	I	I	Icosahedral II	Φ7247PVL/ ΦST5967PVL	H2
LS2183	Zhejinag	ST59	CC59	NT	t437	I	B1	I	I	Icosahedral II	NT	H1
161	Zhejinag	ST188	CC1	NT	t189	I	M	NS	NS	Icosahedral II	NT	H2
SH12	Shanghai	ST88	CC88	NT	t12147	III	A1	IV	III	Icosahedral I	ΦPVL	H2
244	Zhejinag	ST88	CC88	NT	t7637	III	C7	IV	III	Elongated	ΦTCH60	H2
104	Zhejinag	ST88	CC88	NT	t5269	III	N	IV	III	Elongated	ΦTCH60^*^	R2
148	Zhejinag	ST9	CC9	IVb	t309	I	O	VI	II	Icosahedral I	ΦPVL	H2
203	Zhejinag	ST1	CC1	IVa	t127	III	H3	I	NS	Icosahedral I	Φ108PVL^*^	H1
SH9	Shanghai	ST59	CC59	IVa	t441	I	I1	I	I	Icosahedral I	ΦPVL	H1
209	Zhejinag	ST59	CC59	IVa	t437	I	E3	II	I	Elongated	ΦSa2958	H1
LS373	Zhejinag	ST59	CC59	IVa	t1751	I	B3	I	I	Icosahedral II	NT	H2
60	Zhejinag	ST59	CC59	IVa	t437	I	D3	NS	I	Icosahedral II	NT	H2
34	Zhejinag	ST59	CC59	IVa	t437	I	J	I	I	Icosahedral II	NT	H2
65	Zhejinag	ST59	CC59	IVa	t437	I	D1	I	I	Icosahedral II	NT	H2
79	Zhejinag	ST59	CC59	IVa	t437	I	F2	I	I	Icosahedral II	ΦSa119	H2
186	Zhejinag	ST59	CC59	IVa	t437	I	I1	I	I	Icosahedral II	ΦSa119	H2
238	Zhejinag	ST59	CC59	IVa	t437	I	C3	II	I	Icosahedral II	ΦSa119^*^	H2
212	Zhejinag	ST59	CC59	IVa	t437	I	E1	II	I	Elongated	ΦSa2958	H2
226	Zhejinag	ST59	CC59	IVa	t437	I	C3	II	I	Elongated	ΦSa2958	H2
246	Zhejinag	ST338	CC59	IVa	t437	I	C5	II	III	Elongated	ΦTCH60	H2
202	Zhejinag	ST88	CC88	IVa	t7637	III	H1	IV	III	Elongated	ΦTCH60	H2
213	Zhejinag	ST59	CC59	IVa	t1451	I	H1	NS	NS	Icosahedral II	NT	H2
LS1939	Zhejinag	ST59	CC59	Iva	t437	I	B3	I	I	Icosahedral II	NT	H2
SH6	Shanghai	ST59	CC59	IVa	t441	I	A3	I	I	Icosahedral I	ΦPVL	H2
40	Zhejinag	ST59	CC59	IVa	t437	I	J	II	I	Icosahedral II	ΦSa119	R1
218	Zhejinag	ST59	CC59	IVa	t437	NT	E2	II	NS	Icosahedral II	NT	R2
206	Zhejinag	ST59	CC59	IVa	t437	I	H2	II	I	Icosahedral I	ΦPVL	R2
LS2032	Zhejinag	ST59	CC59	III	t437	I	A2	I	I	Icosahedral II	NT	H1
130	Zhejinag	ST59	CC59	III	t441	I	F2	I	I	Icosahedral II	ΦSa119	H1
242	Zhejinag	ST338	CC59	III	t441	I	C2	I	I	Icosahedral II	Φ7247PVL/ ΦST5967PVL	H1
256	Zhejinag	ST59	CC59	III	t5983	I	D2	I	I	Icosahedral II	Φ7247PVL/ ΦST5967PVL	H1
211	Zhejinag	ST22	CC22	III	t5983	I	G1	VI	II	Icosahedral I	Φtp310-1	H1
LS1268	Zhejinag	ST59	CC59	III	t437	I	B3	I	I	Icosahedral II	NT	H1
233	Zhejinag	ST30	CC30	III	t1749	III	L	VII	V	Icosahedral II	NT	H2
219	Zhejinag	ST338	CC59	III	t437	I	C1	I	I	Icosahedral II	NT	H2
82	Zhejinag	ST59	CC59	III	t437	I	F1	I	I	Icosahedral II	NT	H2
253	Zhejinag	ST59	CC59	III	t437	I	D1	I	I	Icosahedral II	NT	H2
8	Zhejinag	ST59	CC59	III	t437	I	D1	I	NS	Icosahedral II	NT	H2
204	Zhejinag	ST59	CC59	III	t437	I	E3	I	I	Icosahedral II	NT	H2
210	Zhejinag	ST59	CC59	III	t437	I	E4	I	I	Icosahedral II	NT	H2
214	Zhejinag	ST22	CC22	III	t5983	I	G1	IV	II	Icosahedral I	ΦPVL	H2
221	Zhejinag	ST338	CC59	III	t437	I	C1	I	I	Icosahedral I	ΦPVL	H2
69	Zhejinag	ST217	CC22	III	t309	I	G3	III	II	Icosahedral I	ΦPVL^*^	H2
195	Zhejinag	ST59	CC59	III	t437	I	P	I	I	Icosahedral II	ΦSa119	H2
51	Zhejinag	ST59	CC59	III	t441	I	D2	I	I	Elongated	ΦSa2958	H2
220	Zhejinag	ST338	CC59	III	t437	I	C1	I	I	Icosahedral II	Φ7247PVL/ ΦST5967PVL	H2
74	Zhejinag	ST59	CC59	III	t437	I	F1	I	I	Icosahedral II	Φ7247PVL/ ΦST5967PVL	H2
170	Zhejinag	ST59	CC59	III	t437	I	F2	I	I	Icosahedral II	Φ7247PVL/ ΦST5967PVL	H2
254	Zhejinag	ST59	CC59	III	t437	I	D1	I	I	Icosahedral II	Φ7247PVL/ ΦST5967PVL	H2
237	Zhejinag	ST59	CC59	III	t437	I	C4	I	I	Icosahedral II	Φ7247PVL/ ΦST5967PVL	H2
236	Zhejinag	ST59	CC59	III	t437	I	C4	I	I	Icosahedral II	Φ7247PVL/ ΦST5967PVL	H2
108	Zhejinag	ST59	CC59	III	t437	I	F2	I	I	Icosahedral II	Φ7247PVL/ ΦST5967PVL	H2
145	Zhejinag	ST59	CC59	III	t437	I	D2	I	I	Icosahedral II	Φ7247PVL/ ΦST5967PVL	H2
217	Zhejinag	ST59	CC59	III	t437	I	E1	I	I	Icosahedral II	Φ7247PVL/ ΦST5967PVL	H2
16	Zhejinag	ST59	CC59	III	t437	I	D3	I	I	Icosahedral II	Φ7247PVL/ ΦST5967PVL	H2
205	Zhejinag	ST22	CC22	III	t5983	I	G1	I	II	Icosahedral I	Φtp310-1	H2
239	Zhejinag	ST22	CC22	III	t5983	I	G2	I	II	Icosahedral I	Φtp310-1	H2
RJ117	Shanghai	ST338	CC59	III	t437	I	A4	I	I	Icosahedral II	NT	H2
LS2137	Zhejinag	ST59	CC59	III	t2755	I	A6	I	I	Icosahedral II	NT	H2
3	Zhejinag	ST59	CC59	III	t034	I	Q	III	III	Elongated	ΦSa2USA	H2
222	Zhejinag	ST149	CC5	II	t437	I	C6	I	I	Elongated	ΦSa2USA	H2
255	Zhejinag	ST149	CC5	II	t437	I	K	II	III	Icosahedral I	Φtp310-1	H2
**MSSA (*****n*** = **16)**												
LS126	Zhejinag	ST30	CC30	–	t318	I	B2	I	I	Icosahedral II	NT	H1
SH14	Shanghai	ST398	CC398	–	t034	I	A5	III	III	Elongated	NT	H1
LS1985	Zhejinag	ST22	CC22	–	t309	I	B1	IV	II	Icosahedral I	ΦPVL	H1
LS2074	Zhejinag	ST25	CC25	–	t227	I	B1	NS	IV	Icosahedral I	ΦPVL	H1
SH26	Shanghai	ST25	CC25	–	t078	I	A2	NS	NS	Elongated	ΦSa2958	H1
SH135	Shanghai	ST1301	CC121	–	t12145	IV	A1	V	VI	Icosahedral I	NT	H1
LS1940	Zhejinag	ST30	CC30	–	t318	III	A7	I	VI	Icosahedral II	NT	H1
LS2078	Zhejinag	ST172	singleton	–	t078	I	B1	NS	VI	Icosahedral I	ΦPVL	H1
LS1004	Zhejinag	ST160	CC121	–	t15797	IV	B3	V	VI	Icosahedral I	NT	H2
LS1966	Zhejinag	ST88	CC88	–	t15796	III	B2	IV	III	Icosahedral I	ΦPVL	H2
SH13	Shanghai	ST88	CC88	–	t2310	III	A3	IV	III	Elongated	ΦTCH60	H2
SH25	Shanghai	ST217	CC22	–	t309	I	A4	VIII	II	Elongated	ΦSa2958	H2
SH19	Shanghai	ST217	CC22	–	t309	I	A4	VIII	II	Elongated	ΦSa2958	H3
SJ1775	Shanghai	ST398	CC398	–	t034	I	I2	III	III	Elongated	ΦSa2USA	R1
SH3	Shanghai	ST398	CC398	–	t1255	I	A3	III	NS	Elongated	ΦSa2USA	R1
LS1911	Zhejinag	ST398	CC398	–	t034	I	B3	III	III	Elongated	ΦSa2USA	R1

* *Phages-like, positive by icosahedral/elongated head classification assays (PCR-1 to -3) and characterization assays (PCR-7 to -15) but negative for linkage assays (PCR-4 and -6). –, no SCCmec elements was detected (MSSA)*.

*NT, non-typeable*.

*NS, could not be sequenced*.

### Typing of PVL-encoding phages

Applying the expanded PCR-based scheme described above, it was possible to identify 10 of known phages carrying *lukSF-PV* genes. Our results showed that 67.9% (53/78, 42 MRSA and 11 MSSA) of PVL-positive isolates could be divided into 8 phage types [ΦPVL (*n* = 12, including one ΦPVL-like), Φ108PVL-like (*n* = 1), Φtp310-1 (*n* = 4), ΦSa2958 (*n* = 7), ΦSa2USA (*n* = 5), ΦTCH60 (*n* = 5, including one ΦTCH60-like), Φ7247PVL/ΦST5967PVL (*n* = 13), and ΦSa119 (*n* = 6, including one ΦSa119-like)], which were almost equally split among elongated-head group (*n* = 17), icosahedral-head group I (*n* = 17), and II (*n* = 19). Phage typeability was 68.8% in MSSA (11 out of 16) and 67.7% in MRSA (42 out of 62). Unfortunately, 25 isolates (32.1%) could not be typed by the present scheme. Four MRSA isolates were considered to be “phage-like,” named ΦPVL-like, Φ108PVL-like, ΦTCH60-like and ΦSa119-like, according to the definition from materials and methods. The details of PVL-encoding phages types were shown in Table 2.

### Variation of *lukSF-PV* genes

The amplification products of the *lukSF-PV* genes from 78 isolates were sequenced, and nucleotide variations were seen at five sites (position 527 and 663 located in the *lukS* locus and position 1022, 1396, 1729 located in the *lukF* locus) using the *lukSF-PV* genes of ΦSLT as a reference. Of 78 PVL-positive isolates, 71 (91.0%, 71/78) were of H variant as defined by O'Hara et al. (2008), which can be further separated into H1 (Genbank Accession no. EF571669) (23.1%, 18/78), H2 (Genbank Accession no. EF571668) (66.7%, 52/78), and H3 (Genbank Accession no. EF571713) (1.3%, 1/78) groups differed at nucleotide position 1396 and 663 (Table 2). The rest of seven isolates were identified as R variant displaying non-synonymous nucleotide 527 A to G mutation, and further divided into R1 (Genbank Accession no. EF571829) (5.1%, 4/78), and R2 (Genbank Accession no. EF571830) (3.8%, 3/78) groups according to the distinction of nucleotide site 1729 (Table 2).

### DNA sequencing of phage/chromosome junctions

**Figure 3:**
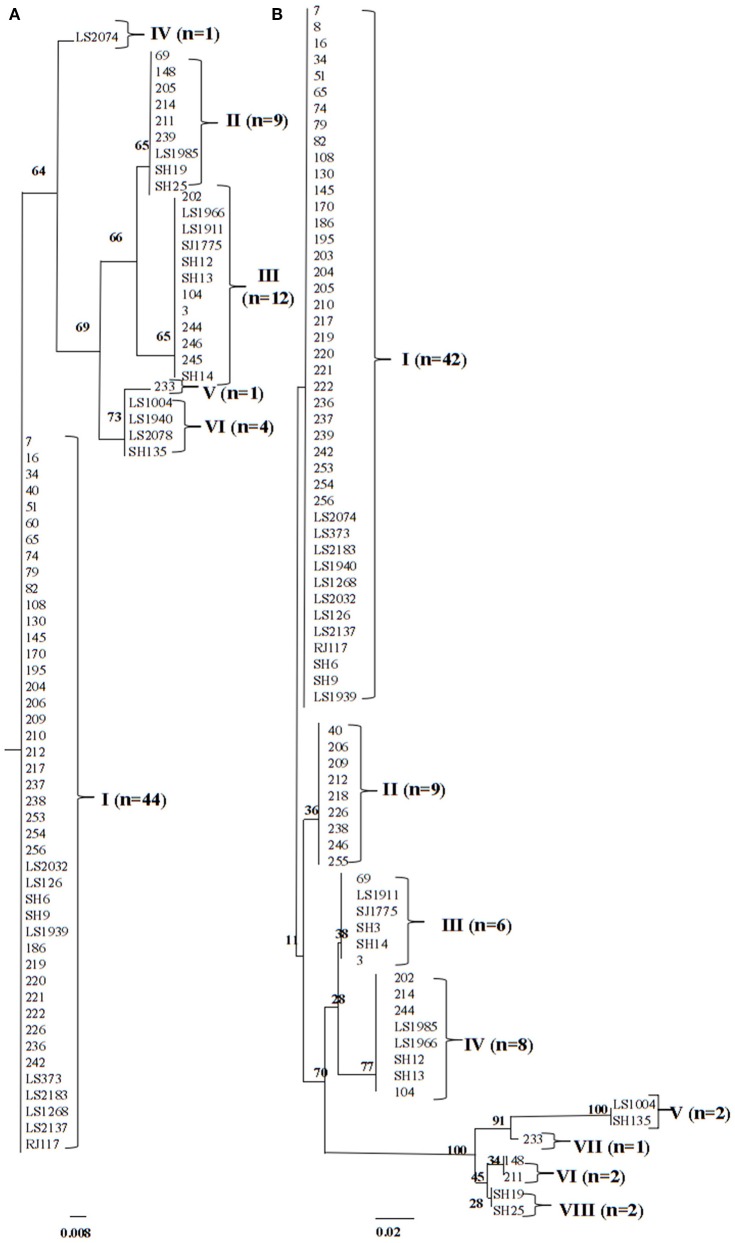
**ML analysis trees for (A) ***attR*** attachment site (71 isolates) and (B) ***attL*** attachment site (72 isolates)**. Among 78 PVL-positive isolates, the *attR* and *attL* attachment site sequences cannot be identified in 7 and 6 strains, respectively. Branching numbers represent bootstrap values.

To investigate the chromosomal insertion site of phage in the present study, we performed PCR based on the known phage/chromosome junctions in the other strains (Boakes et al., 2011; Li et al., 2011). Seventy two (92.3%) isolates showed positive amplification for *attL* sequences, and 71 (91.0%) for *attR* sequences. This indicated a relatively conserved PVL-encoding phage integration site regardless of difference among genetic features of strains, PVL-encoding phage types, and *lukSF-PV* genes variants. Maximum-likelihood phylogenetic tree analysis of the *attR* and *attL* attachment sites showed four clusters (I, II, III, and VI) and two singletons (IV and V) for *attR* and seven clusters (I, II, III, IV, V, VI, and VIII) and one singleton (VII) for *attL* (Figure 3, Table 2).

## Discussion

PVL-positive *S. aureus*, strongly associated with SSTI and necrotizing pneumonia, has gained great attention in recent years (David and Daum, 2010; Shallcross et al., 2013). In China, the PVL positive rate ranged from 5.5 to 28.6% in HA-MRSA isolates (Yu et al., 2008; Fan et al., 2009; Li et al., 2012; Xiao et al., 2013; Hu et al., 2015). The major clone with *lukSF-PV* genes was ST59 in MRSA, while ST398, and ST88 in MSSA according to the previous studies (Yu et al., 2008; Fan et al., 2009; Li et al., 2012; Xiao et al., 2013; Hu et al., 2015). In the current study, we also revealed a high percentage of PVL-positive ST59 clones associated with MRSA and ST398 with MSSA strains.

A previously published eight-reaction PCR assay was performed to identify five (ΦSa2958, ΦSa2mw, ΦPVL, Φ108PVL, and ΦSLT) of the PVL-encoding phages in *S. aureus* (Ma et al., 2008). Subsequently, this scheme was improved to include the detection of ΦSa2USA (Boakes et al., 2011), ΦTCH60, Φtp310-1, and Φ7247PVL (Chen et al., 2013). Recently, Sanchini et al. (2014) divided PVL-encoding phages into three groups including the elongated-head group, icosahedral-head group I, and icosahedral-head group II, and incorporated ΦSa119 and ΦST5967PVL belonging to icosahedral-head group II into the scheme. However, this PCR-based scheme could not discriminate ΦST5967PVL from Φ7247PVL (Chen et al., 2013; Sanchini et al., 2014), because the genomes of both phages showed more than 99% identity (Zhang et al., 2011). Therefore, these two phages were marked as “Φ7247PVL/Φ5967PVL” in this study.

Applying the PCR-based strategy, the distribution of PVL-encoding phages was investigated all over the world. Two morphologically distinct phages (ΦPVL and ΦSa2958) were found to be predominant among Japanese PVL-positive MRSA (Ma et al., 2008), and ΦSa2USA was the most prevalent phage in Italy (Sanchini et al., 2014), while in United Kingdom, the most common phage types were ΦSa2USA and ΦSa2mw (Otter et al., 2010; Boakes et al., 2011). In this study, the results revealed that Φ7247PVL/ΦST5967PVL, and ΦPVL were the major PVL phage types in the isolates studied. Notably, all the untypeable isolates were positive for PCR-3 and belonged to ST30 and ST59 strains, indicating that both the clones may harbor novel unknown icosahedral-head group II phage type(s) in China. In line with the previous observations (Ma et al., 2008; Otter et al., 2010; Boakes et al., 2011; Chen et al., 2013; Sanchini et al., 2014), lineage-specificity of PVL-encoding phages were noted in this study. For instance, 92.3% of Φ7247PVL/ΦST5967PVL-carrying isolates belonged to CC59 lineage, harboring cluster I *attR*, and *attL*, and 60% of ΦSa2USA-hosting isolates pertained to ST398. However, the ΦPVL-carrying isolates showed more variability of genetic characterization, with CC22, CC25, CC59, CC88, and CC9 detected within this group. ΦSa119, recently identified in CC5 lineage by Sanchini et al. (2014), was detected in CC59 lineage. ΦSa2958, associated with MRSA belonging to CC30 (Ma et al., 2008), was related to CC22 and CC59 in the present study.

These findings are similar with those reported in China previously (Li et al., 2012; Hu et al., 2015). However, Φ108PVL, which was the predominant phage type in CA-MRSA from children in mainland China (Li et al., 2012), and ΦSLT, which was prevalent in 74 health care-associated PVL-positive MRSA strains (Hu et al., 2015), are not common in the present study. The difference of regions across China and sources of isolates may account for the dissimilarity of the predominant PVL-encoding phages among these studies.

The correlation between the presence of PVL in *S. aureus* infections and the clinical outcome has been controversial because of some conflicting data from epidemiological study or *in vivo* animal models (Hermos et al., 2010; Otto, 2011). Corresponding protein models of two *lukSF-PV* sequence variants, named R and H variant, may indicate certain functional significance (Wolter et al., 2007; John and Lindsay, 2008; O'Hara et al., 2008). In our study, the H- and R-PVL isoforms could be identified in three morphological phage groups. Of note, the R1 isoform was mainly associated with ΦSa2USA, similar to the previous study (Chen et al., 2013). Of the five SNPs in the present study, two were non-synonymous, including a previously described arginine to histidine replacement at amino acid residue 176 (nucleotide 527) and a valine to isoleucine replacement at amino acid 340 (nucleotide 1022). The latter amino acid change presented in only one sequence (LS2074) and decreased the predicted molecular size of LukF from 36962Da to 36948Da, but did not influence the theoretical isoelectric point (9.1). The nucleotide sequence of the *lukSF-PV* genes has been deposited in GenBank under the Accession no. KX443594. Further work is required to confirm whether this non-synonymous replacement results in clinical significance.

Another interesting finding was that all the *attR* cluster I-harboring isolates possessed the same *agr* type (*agr* I) and were mostly observed within CC59 lineage. With the exception of one strain (strain 148), all of the *attR* sequences of cluster II strains were identified in CC22 lineage. The CC88 isolates were completely associated with *attR* cluster III and *attL* cluster IV. The main variations in *attL* were located in the phage binding sites (P1 and P2) and those in *attR* were located in the phage binding site P3 and chromosome binding sites B2. These indicated that the diversity of junction sequences was mainly due to differences in the phage DNA, which was different from the previous report that the main variations in *attR* were located in the 29-bp chromosome binding sites (B2) (Chen et al., 2013).

There were two limitations in this study. First, the identification of PVL-encoding phages was on the basis of PCR assays targeting only a part of the phage genome. Since phages tend to have recombination events, positivity by PCR should be supposed to be a family of PVL-encoding phages with variable genomic portions, instead of being taken as a specific phage (Sanchini et al., 2014; Xia and Wolz, 2014). Second, the proportion of phage-untypable PVL-positive isolates is a little high (32.1%), although the rate is within the range of 16.4–77.6% reported previously (Ma et al., 2008; Boakes et al., 2011; Li et al., 2012; Hu et al., 2015) by the PCR-based scheme. This could be because some unknown or newly published PVL-encoding phages such as Φ7401PVL (Mariem et al., 2013) have not being included in the scheme. Therefore, unknown PVL-encoding phages remain to be discovered and new primer pairs are expected to be designed to further expand this scheme. Whatever, next-generation sequencing may provide us with a way to achieve perfect epidemiological picture of diversity of PVL-encoding phages, which seems to represent a trend in the future.

## Conclusion

This study characterized PVL-encoding phages, the chromosomal phage insertion sites, the polymorphism of *lukSF-PV* genes, and the genetic background of PVL-positive *S. aureus* clinical isolates from China, and found the existence of some correlation among them. Our findings may contribute to the understanding of the epidemiology and evolution of PVL-positive *S. aureus*, and add the evidence that PVL-positive strains disseminating worldwide likely carry distinct PVL phages.

## Author contributions

HZ, FH performed the experiments; QL designed and conceived the study; HZ, QL, CH analyzed the clinical data and wrote this manuscript; QL, SJ, XX, YZ, BD, and FG collected the clinical samples. All authors read and approved the final manuscript.

### Conflict of interest statement

The authors declare that the research was conducted in the absence of any commercial or financial relationships that could be construed as a potential conflict of interest.

## References

[B1] Bignardi G. E. Woodford N. Chapman A. Johnson A. P. Speller D. C. (1996). Detection of the *mec-A* gene and phenotypic detection of resistance in *Staphylococcus aureus* isolates with borderline or low-level methicillin resistance. J. Antimicrob. Chemother. 37, 53–63. 10.1093/jac/37.1.53 8647774

[B2] Boakes E. Kearns A. M. Ganner M. Perry C. Hill R. L. Ellington M. J. (2011). Distinct bacteriophages encoding Panton-Valentine leukocidin (PVL) among international methicillin-resistant *Staphylococcus aureus* clones harboring PVL. J. Clin. Microbiol. 49, 684–692. 10.1128/JCM.01917-10 21106787 PMC3043515

[B3] Boyle-Vavra S. Daum R. S. (2007). Community-acquired methicillin-resistant *Staphylococcus aureus*: the role of Panton-Valentine leukocidin. Lab. Invest. 87, 3–9. 10.1038/labinvest.3700501 17146447

[B4] Chen L. Chavda K. D. Solanki M. Mediavilla J. R. Mathema B. Schlievert P. M.. (2013). Genetic variation among Panton-Valentine leukocidin-encoding bacteriophages in *Staphylococcus aureus* clonal complex 30 strains. J. Clin. Microbiol. 51, 914–919. 10.1128/JCM.03015-12 23284024 PMC3592069

[B5] Clinical Laboratory Standards Institute (2014). Performance Standards for Antimicrobial Susceptibility Testing; Twenty-Second Informational Supplement (M100-S22), Vol. 34 Wayne, PA: Clinical and Laboratory Standards Institute.

[B6] David M. Z. Daum R. S. (2010). Community-associated methicillin-resistant *Staphylococcus aureus*: epidemiology and clinical consequences of an emerging epidemic. Clin. Microbiol. Rev. 23, 616–687. 10.1128/CMR.00081-09 20610826 PMC2901661

[B7] Enright M. C. Day N. P. Davies C. E. Peacock S. J. Spratt B. G. (2000). Multilocus sequence typing for characterization of methicillin-resistant and methicillin-susceptible clones of *Staphylococcus aureus*. J. Clin. Microbiol. 38, 1008–1015. 10.1128/JCM.43.9.4448-4454.2005 10698988 PMC86325

[B8] Fan J. Shu M. Zhang G. Zhou W. Jiang Y. Zhu Y.. (2009). Biogeography and virulence of *Staphylococcus aureus*. PLoS ONE 4:e6216. 10.1371/journal.pone.0006216 19593449 PMC2705676

[B9] Ganesan A. Crawford K. Mende K. Murray C. K. Lloyd B. Ellis M.. (2013). Evaluation for a novel methicillin resistance (*mecC*) homologue in methicillin-resistant *Staphylococcus aureus* isolates obtained from injured military personnel. J. Clin. Microbiol. 51, 3073–3075. 10.1128/JCM.01516-13 23784136 PMC3754630

[B10] Hermos C. R. Yoong P. Pier G. B. (2010). High levels of antibody to Panton-Valentine leukocidin are not associated with resistance to *Staphylococcus aureus*-associated skin and soft-tissue infection. Clin. Infect. Dis. 51, 1138–1146. 10.1086/656742 20946065 PMC2962716

[B11] Hu Q. Cheng H. Yuan W. Zeng F. Shang W. Tang D.. (2015). Panton-Valentine leukocidin (PVL)-positive health care-associated methicillin-resistant *Staphylococcus aureus* isolates are associated with skin and soft tissue infections and colonized mainly by infective PVL-encoding bacteriophages. J. Clin. Microbiol. 53, 67–72. 10.1128/JCM.01722-14 25339405 PMC4290966

[B12] John J. F. Jr. Lindsay J. A. (2008). Clones and drones: do variants of Panton-Valentine leukocidin extend the reach of community-associated methicillin-resistant *Staphylococcus aureus*? J. Infect. Dis. 197, 175–178. 10.1086/524693 18177251

[B13] Koreen L. Ramaswamy S. V. Graviss E. A. Naidich S. Musser J. M. Kreiswirth B. N. (2004). *spa* typing method for discriminating among *Staphylococcus aureus* isolates: implications for use of a single marker to detect genetic micro- and macro variation. J. Clin. Microbiol. 42, 792–799. 10.1128/JCM.42.2.792-799.2004 14766855 PMC344479

[B14] Li X. Sun J. Wu D. Wang L. Yang Y. Wang C.. (2012). Panton-Valentine leukocidin gene sequence variation and phage in methicillin-resistant and methicillin-susceptible *Staphylococcus aureus* from children in mainland China. Microbiol. Immunol. 56, 155–162. 10.1111/j.1348-0421.2011.00422.x 22469181

[B15] Li Z. Stevens D. L. Hamilton S. M. Parimon T. Ma Y. Kearns A. M.. (2011). Fatal *S. aureus* hemorrhagic pneumonia: genetic analysis of a unique clinical isolate producing both PVL and TSST-1. PLoS ONE 6:e27246. 10.1371/journal.pone.0027246 22110621 PMC3207839

[B16] Lina G. Boutite F. Tristan A. Bes M. Etienne J. Vandenesch F. (2003). Bacterial competition for human nasal cavity colonization: role of Staphylococcal *agr* alleles. Appl. Environ. Microbiol. 69, 18–23. 10.1128/AEM.69.1.18-23.2003 12513972 PMC152380

[B17] Lina G. Piémont Y. Godail-Gamot F. Bes M. Peter M. O. Gauduchon V.. (1999). Involvement of Panton-Valentine leukocidin-producing *Staphylococcus aureus* in primary skin infections and pneumonia. Clin. Infect. Dis. 29, 1128–1132. 10.1086/313461 10524952

[B18] Ma X. X. Ito T. Kondo Y. Cho M. Yoshizawa Y. Kaneko J.. (2008). Two different Panton-Valentine leukocidin phage lineages predominate in Japan. J. Clin. Microbiol. 46, 3246–3258. 10.1128/JCM.00136-08 18685010 PMC2566072

[B19] Mariem B. J. Ito T. Zhang M. Jin J. Li S. IIhem B. B.. (2013). Molecular characterization of methicillin-resistant Panton-valentine leukocidin positive *Staphylococcus aureus* clones disseminating in Tunisian hospitals and in the community. BMC Microbiol. 13:2. 10.1186/1471-2180-13-2 23289889 PMC3544733

[B20] Mulvey M. R. Chui L. Ismail J. Louie L. Murphy C. Chang N.. (2001). Development of a Canadian standardized protocol for subtyping methicillin-resistant *Staphylococcus aureus* using pulsed-field gel electrophoresis. J. Clin. Microbiol. 39, 3481–3485. 10.1128/JCM.39.10.3481-3485.2001 11574559 PMC88375

[B21] O'Hara F. P. Guex N. Word J. M. Miller L. A. Becker J. A. Walsh S. L.. (2008). A geographic variant of the *Staphylococcus aureus* Panton-Valentine leukocidin toxin and the origin of community-associated methicillin-resistant *S. aureus* USA300. J. Infect. Dis. 197, 187–194. 10.1086/524684 18177252

[B22] Otter J. A. Kearns A. M. French G. L. Ellington M. J. (2010). Panton-Valentine leukocidin-encoding bacteriophage and gene sequence variation in community-associated methicillin-resistant *Staphylococcus aureus*. Clin. Microbiol. Infect. 16, 68–73. 10.1111/j.1469-0691.2009.02925.x 19709067

[B23] Otto M. (2011). A MRSA-terious enemy among us: end of the PVL controversy? Nat. Med. 17, 169–170. 10.1038/nm0211-169 21297612

[B24] Paulander W. Nissen V. A. Bæk K. T. Haaber J. Frees D. Ingmer H. (2013). Antibiotic-mediated selection of quorum-sensing-negative *Staphylococcus aureus*. mBio 3:e00459-12. 10.1128/mBio.00459-1213 23143800 PMC3509436

[B25] Sanchini A. Del G. M. Villa L. Ammendolia M. G. Superti F. Monaco M.. (2014). Typing of Panton-Valentine leukocidin-encoding phages carried by methicillin-susceptible and methicillin-resistant *Staphylococcus aureus* from Italy. Clin. Microbiol. Infect. 20, O840–O846. 10.1111/1469-0691.12679 24835735

[B26] Shallcross L. J. Fragaszy E. Johnson A. M. Hayward A. C. (2013). The role of the Panton-Valentine leucocidin toxin in staphylococcal disease: a systematic review and meta-analysis. Lancet Infect. Dis. 13, 43–54. 10.1016/S1473-3099(12)70238-4 23103172 PMC3530297

[B27] Uhlemann A. C. Otto M. Lowy F. D. DeLeo F. R. (2014). Evolution of community- and healthcare-associated methicillin-resistant *Staphylococcus aureus*. Infect. Genet. Evol. 21, 563–574. 10.1016/j.meegid.2013.04.030 23648426 PMC3884050

[B28] Wolter D. J. Tenover F. C. Goering R. V. (2007). Allelic variation in genes encoding Panton-Valentine leukocidin from community-associated *Staphylococcus aureus*. Clin. Microbiol. Infect. 13, 827–830. 10.1111/j.1469-0691.2007.01763.x 17610602

[B29] Xia G. Wolz C. (2014). Phages of *Staphylococcus aureus* and their impact on host evolution. Infect. Genet. Evol. 21, 593–601. 10.1016/j.meegid.2013.04.022 23660485

[B30] Xiao M. Wang H. Zhao Y. Mao L. L. Brown M. Yu Y. S.. (2013). National surveillance of methicillin-resistant *Staphylococcus aureus* in China highlights a still-evolving epidemiology with 15 novel emerging multilocus sequence types. J. Clin. Microbiol. 51, 3638–3644. 10.1128/JCM.01375-13 23985906 PMC3889745

[B31] Yu F. Chen Z. Liu C. Zhang X. Lin X. Chi S.. (2008). Prevalence of *Staphylococcus aureus* carrying Panton-Valentine leukocidin genes among isolates from hospitalised patients in China. Clin. Microbiol. Infect. 14, 381–384. 10.1111/j.1469-0691.2007.01927.x 18190580

[B32] Zhang K. McClure J. A. Elsayed S. Louie T. Conly J. M. (2005). Novel multiplex PCR assay for characterization and concomitant subtyping of staphylococcal cassette chromosome *mec* types I to V in methicillin-resistant *Staphylococcus aureus*. J. Clin. Microbiol. 43, 5026–5033. 10.1128/JCM.43.10.5026-5033.2005 16207957 PMC1248471

[B33] Zhang M. Ito T. Li S. Jin J. Takeuchi F. Lauderdale T. L.. (2011). Identification of the third type of PVL phage in ST59 methicillin-resistant *Staphylococcus aureus* (MRSA) strains. FEMS Microbiol. Lett. 323, 20–28. 10.1111/j.1574-6968.2011.02355.x 21732964

